# Austin District Medical Society

**Published:** 1893-04

**Authors:** 


### AUSTIN DISTRICT MEDICAL SOCIETY.

---

There was a fair attendance of members of the Austin District Medical Society at the 22nd quarterly meeting which was held in Austin on the 23rd of March ult. Dr. R. P. Talley of Temple,

President, in the chair, and Secretary Dr. T. J. Bennett at the desk. Representatives from Belton, Bartlet, Burnett, Temple, Waco, Elgin and other places were present, and two lady physicians graced the hall with their presence, Doctor Leake, and Dr. Holder. Dr. J. M. Strayhorn of Bartlett joined the society. Dr. Caldwell of Waco was a visitor.

Dr. G. W. Christian, retiring president, delivered his address, which was, on motion, [illegible]rred to the Publishing Committee, and will appear in the jou[illegible]

HEMIPLEGIA CHANGED INTO PARAPLEGIA:—Dr. Sam Cunningham of Elgin presented before the society a little girl of thirteen years of age who had, presumably as the result of a fall, suffered an attack of fever, on the third day of which partial hemiplegia made its appearance, the right side being affected. This occurred in July '92. The paralysis was confined to the arm and leg, the face and tongue (tongue only slightly deflected) not participating, nor the eyelids. In four weeks the paralysis had nearly left the right arm, and the left leg became paralyzed; the case now being one of paraplegia. Dr. Cunningham asked for a diagnosis. All the physicians present examined the child and discussed the probable cause and pathology, the preponderance of opinion being, in favor of spinal pressure. Dr. Poyner thought it was likely a case of peripheral reflex, in as much, he said, as if caused by spinal pressure, the paralysis wonld have been fixed, whereas it seemed to shift about; but he was not prepared to say whether the genitalia, or the digestive tract, or what not was the seat of the irritation; on the other hand Dr. Christian called attention to the clonic spasmodic contraction of the toes; a symptom, he said, never present in paralysis resulting from reflex irritation. The eyes were not affected. Dr. McLaughlin inclined to a belief that it was a multiple neuritis. All concurred in the opinion that the case should be treated "on general principles." Dr. Cunningham explained that he had exhausted his resources, empirically, of course; had used counter irritation, electricity, strychnia, and bitter tonics, iron etc. Extension was suggested by Dr. Caldwell, in which Dr. Bennett concurred; and Dr. Davis advised iodide of potassium. The child appeared to be fairly well nourished and in pretty good general health; mental faculties unimpaired.

Dr. F. S. White, Supt. S. Lunatic Asylum, read a paper entitled "Can nerve action be accounted for by McLaughlin's physical theory?" Discussed by Drs. Denton, McLaughlin and others and referred to publishing committee.

A Point in Legal Medicine.—Dr. Talley read a transcript of the evidence in the case of State *vs*. Geo. Fowler, on charge of rape. At last term of Bell county court Geo. Fowler white, aged 25, a discharged lunatic, known to possess the criminal impulse, was convicted of rape upon a white woman; in fact he made a full con-fession. The testimony was conclusive, both as to the act and to his insanity. Nevertheless he was sentenced to the penitentiary for life; the *justification* with the jury b[illegible] protection of the people of Bell county. Fowler had bee[illegible] for attempt at train wrecking, some years ago, and was acquitted on grounds of insanity, and was sent to the asylum. The then Superintendent, Dorsett, furloughed him, and as he did not return, he was discharged. When Dr. Reeves took charge of the Asylum, Fowler was recorded as discharged in default of returning from furlough, and so the record stands to-day. The Bell county people say that if he were sent to the Asylum he would be turned loose again, and so the jury sent him to the penitentiary. The point presented for discussion was, "is a jury justified in convicting a lunatic? and the court in sentencing him to the penitentiary?"

The subject was extensively discussed, without any conclusion being reached. Dr. Davis offered a resolution to the effect that it is the sense of the A. D. M. S. that lunatics with erotic mania should be castrated as a therapeutic measure. Resolution was tabled. It was agreed that the paper be left with the editor of Daniel's Texas Medical Journal and made the basis of a paper for the Medico Legal Congress, which is soon to meet in Chicago.

***

What to do with the criminal insane in this State is a serious problem in view of the fact that the number of insane largely exceeds the accommodation for them; and to provide asylum and treatment for the more recent (and supposed, curable) cases, the law is such that cases of less than one year standing shall have precedence in admittance; and to make room for them, the chronic cases are sometimes injudiciously discharged, or furloughed. It is a custom with Superintendents to furlough a lunatic, and if he does not return he is discharged; and in his absence the asylum is filled by acute cases, and there is no room for him; he cannot get back. Hence, dangerous lunatics who ought to be confined, are at large, and a standing menace to the community. The man Fowler was dreaded; it was thought he would commit some crime. Henry Purnell, who killed Superintendent Reeves, Dec., '91, was furloughed. He is lying in jail now, because, as it is alleged, there is no room for him in the asylums, preference being given to cases of less than a year's standing. The laws badly need amending. Provision must and should be made for this class; there is none. They are either at large, or in jail, many of them. Some provision should be made at the Asylum for keeping this class confined, and separated from all others. Humanity revolts at sentencing an irresponsib[illegible]erson to the penitentiary for life, but what are we to do? Th[illegible]e as dangerous as if they *were* responsible, and more so. Having the brute instinct, and no moral sense, they are as dangerous as wild beasts.

***

The Austin District Medical Society numbering 80 active members, is entitled to sixteen delegates to the State Medical Association's annual meeting which will convene in Galveston May 2, prox. The following have been appointed:

Dr. F. R. Martin, of Kyle.
Dr. J. F. Dean, of Hornsby.
Dr. J. S. Poyner, of Bartlett.
Dr. H. H. Thorpe, of Liberty Hill.
Dr. A. Garwood, of New Braunfels.
Dr. Sam Cunningham, of Elgin.
Dr. J. S. Brown, of Taylor.
Dr. R. P. Talley, of Temple.
Dr. W. T. Richmond, of Manor.
Dr. C. A. Danforth, of Granger.
Dr. F. E. Daniel, of Austin.
Dr. R. M. Swearingen, of Austin.
Dr. A. N. Denton, of Austin.
Dr. J. W. McLaughlin, of Austin.
Dr. Mat Smith, of Austin.
Dr. T. J. Tyner, of Austin.
Dr. S. E. Hudson, of Austin.

---

Be sure to go to Galveston May 2. See program—the best in years. Strawberries and soft shell crabs are now in season, and the city is just lovely. Excursions on the bay and down to the oyster beds are part of the fun in store. Papers will be read by distinguished men from abroad. Amongst them Price of Philadelphia, and Cartledge and McMurtry, of Louisville. Prof. Cain, of Nashville, is expected also. It will be one of the biggest Texas turnouts the doctors ever had.

